# The role of the dentist in promoting HPV vaccination in adults: a systematic review and meta-analysis

**DOI:** 10.1186/s12903-026-08517-x

**Published:** 2026-07-16

**Authors:** Ana Rita Fernandes Teixeira, José Frias-Bulhosa, Juan Antonio Ruiz Roca, Otília Pereira-Lopes

**Affiliations:** 1 Faculty of Health Sciences, FP-I3ID, Fernando Pessoa University, Porto, Portugal; 2 Faculty of Medicine, University of Murcia, Murcia, España; 3 IPO Porto Research Center (CI-IPO Porto), Portuguese Oncology Institute of Porto (IPO Porto), Porto, Portugal; 4 RISE - Health Research Network, Porto, Portugal

**Keywords:** Human papillomavirus, HPV, Vaccination, Health education, Adults, Dental health services

## Abstract

**Background:**

Oral health professionals may play a key role in promoting HPV vaccination among adults through counseling and health education. Understanding their impact and the effectiveness of educational interventions is crucial for public health strategies.

**Objectives:**

To assess the influence of oral health professionals on adherence to HPV vaccination in adults and to identify the effectiveness of educational strategies aimed at improving their counseling practices.

**Eligibility criteria:**

This review included cross-sectional and interventional studies evaluating adult-focused HPV vaccination counseling provided by oral health professionals.

**Information sources:**

Searches were conducted in PubMed, EBSCO, the Cochrane Library, Science Direct, Wiley, and TRIP Database from inception to April 21, 2025.

**Methods of synthesis:**

Data were analyzed using a random-effects meta-analysis model in RStudio (version 4.2.2) to estimate pooled proportions with 95% confidence intervals (CIs).

**Results:**

A total of 10 studies were included, involving 9,575 participants, of which 3 studies implemented educational interventions. Prior to educational interventions, many dentists demonstrated limited knowledge about HPV and its link to oropharyngeal cancer, and only a few routinely discussed HPV vaccination with patients. Post-intervention, there was a notable improvement in professionals' knowledge, confidence, and frequency of patient counseling.

**Discussion:**

Educational interventions were effective in enhancing dentist's preparedness to address HPV vaccination. However, barriers such as lack of privacy, discomfort discussing the topic, and limited prior training were also reported.

**Limitations:**

Heterogeneity in study design, limited geographic diversity, and variability in intervention approaches were noted.

**Conclusions:**

Oral health professionals can significantly contribute to adult HPV vaccination efforts through targeted counseling, especially when supported by ongoing education and institutional strategies that address systemic barriers.

**Registration:**

PROSPERO ID: CRD42024617902.

**Supplementary Information:**

The online version contains supplementary material available at https://doi.org/10.1186/s12903-026-08517-x.

## Introduction

The global rise in HPV-associated oropharyngeal cancers, particularly in high-income countries where traditional risk factors such as tobacco use have declined, marks a significant shift in the epidemiology of these malignancies, with HPV now identified as a leading cause [1, 2]. This trend highlights that traditional cancer prevention strategies, such as tobacco control, are insufficient for this specific malignancy, underscoring a critical public health challenge that necessitates urgent preventive measures. Expanding vaccination efforts through the integration of non-traditional settings, such as dental clinics, is essential to enhance vaccine uptake and reduce the incidence of HPV-related diseases, including cervical, anal, and oropharyngeal cancers [3, 4].

Despite substantial global efforts to implement vaccination programs, progress remains uneven. Seventeen years after initial approval of the HPV vaccine, only 55% of the World Health Organization (WHO) Member States have incorporated it as a primary prophylaxis measure, and only 33 out of those 107 countries offer gender-neutral programs that include all adolescents, regardless of sex or gender identity [5, 6]. In Europe, 77% of countries have introduced the HPV vaccination, typically through national immunization programs. However, implementation timelines vary significantly across nations, reflecting different public health priorities and resource allocations [7].

Oral health professionals (OHPs), including dentists and hygienists, occupy a unique position in promoting public health due to their regular access to the oral cavity and consistent interactions with patients during routine care [8]. In recent years, the scope of dental practice has increasingly incorporated preventive health discussions, providing opportunities to address topics such as HPV vaccination, particularly in relation to oropharyngeal cancer, a condition whose prevention is often undervalued [9]. In 2019, 65.5% of adults aged 18–64 in the United States reported a dental visit in the previous 12 months, which may have been with a dentist, a hygienist, or another OHP [10].

Notably, in 2018 approximately 28.2 million Americans (8.3% of the population) visited a dental professional without attending a medical visit, representing a unique opportunity to reach individuals who may have limited interaction with primary care [11]. Despite the strategic access OHPs have to patients, this potential remains largely untapped in the face of suboptimal adult vaccination rates, which are driven by misinformation, myths about vaccine safety, sociocultural barriers, and inconsistent provider recommendations [12]. For example, data from the 2018 United States National Health Interview Survey (NHIS), a nationally representative study of adults aged 19–45, showed that the estimated proportion receiving at least one dose of HPV vaccine was 36.3% (females: 51.5%, males: 21.2%, *p* < 0.001), with even lower rates among mid-adults [(defined as individuals aged 27 to 45 years, aligning with the NHIS age categorization and current FDA/CDC catch-up vaccination guidelines) (females: 15.8%, males: 3.2%, *p* < 0.001)] [13]. The persistence of sex-based disparities reflects the historical prioritization of female vaccination and highlights the need for targeted strategies addressing all genders.

Given their strategic position, OHPs can play a critical role in bridging gaps in HPV vaccination coverage, especially for populations at higher risk for HPV-associated oropharyngeal cancers. Evidence indicates that many unvaccinated individuals visit dental practices annually, creating opportunities for OHPs to provide educational recommendations on vaccination [14]. Notably, data from 2018 show that approximately 28.2 million Americans (8.3% of the population) visited a dental professional without attending a medical visit, representing a substantial subset of the population that could be reached for vaccination counseling through the dental setting ecosystem.

Although several individual studies have examined the role of OHPs in promoting HPV-related health, few comprehensive and up-to-date reviews have synthesized their effectiveness in increasing vaccination rates among adults. Previous reviews have often focused on adolescent vaccination [15] or on health care providers in general, with limited emphasis on dental professional [16, 17]. Furthermore, earlier syntheses, such as Walker et al. [18], predate the COVID-19 pandemic, a global health crisis that substantially altered public perceptions of vaccination. Despite the widespread acceptance of COVID-19 vaccines, HPV vaccination rates among adults remain disproportionately low, suggesting that hesitancy is influenced by distinct factors that require updated analysis in the post-pandemic context. More recently, Chan et al. [19] explored the perspectives of OHPs and students regarding HPV-related knowledge, attitudes, and behaviors, their review primarily focused on provider perspectives, with limited attention to how adult patients perceive and respond to HPV vaccination counseling in dental settings.

To address these gaps, this systematic review and meta-analysis aims to evaluate the role of OHPs in promoting HPV vaccination in adults, identify barriers to their involvement, assess the impact of education interventions, and highlight their potential to enhance vaccine uptake through oral healthcare services.

## Materials and methods

### Protocol registration

This systematic review and meta-analysis were conducted following the PRISMA 2020 guidelines [20].

The research protocol was prospectively registered in the International Prospective Register of Systematic Review (PROSPERO), under ID: CRD42024618902, to ensure transparency and reproducibility.

### Eligibility criteria

This review included studies that examined the role of OHPs in promoting HPV vaccination among adults. Eligible participants comprised dentists, dental hygienists, dentistry and dental hygiene students and adult patients aged 18 years or older. The inclusion of students aimed to capture the impact of early education and training on preparedness to counsel patients, while adult patients were included to assess their receptiveness to HPV vaccination advice delivered in dental settings.

Given the exploratory and public health-oriented nature of this review, the PICO framework was intentionally expanded to reflect the dental setting ecosystem, ensuring a comprehensive overview of vaccination-related perspectives within this unique environment. The PICO framework was defined as follows:

- Population (P): Oral health professionals (dentist, dental hygienists, and students) and adult patients (≥ 18 years)
- Intervention (I): HPV vaccination counseling provided by oral health professionals and students to adults
- Comparison (C): Absence of such counseling
- Outcome (O): Adoption of a positive attitude towards HPV vaccination by adults

Studies were included if they involved adult participants (vaccinated or unvaccinated), were available in full text, and published in English, Portuguese, or Spanish. Studies focusing on other health professionals (e.g., physicians, nurses), child or adolescent populations, or publication types such as letters to the editor, reviews, meta-analyses, and opinion pieces were excluded.

Studies exclusively addressing ‘‘intention to recommend’’ HPV vaccination without evaluating actual counseling practices or educational interventions were also excluded to maintain focus on tangible behaviors and outcomes.

### Information sources and search strategy

A comprehensive literature search was performed across multiple databases: PubMed, the Cochrane Library, TRIP database, ScienceDirect, SciELO, Google Scholar and the Institutional Repository of Fernando Pessoa University. The search was limited to articles published within the last 10 years, in English, Portuguese and Spanish.

The search terms included MeSH and keyword combinations related to oral health, dental health, oral hygiene, care, prevention, HPV, human papillomavirus, education, awareness, health promotion and health education, combined using Boolean connectors ‘‘AND’’, ‘‘OR’’ (Table 1). The final search was conducted on April 21 st, 2025.

**Table 1:** Search strategies used in the databases. Presentation of the terms used, Boolean operators and filters applied in each database to identify studies relevant to the theme of promoting HPV vaccination by oral health professionals

Database	Search Stratagy	Nº Articles
PubMed	((HPV vaccination) OR (human papillomavirus vaccine)) AND ((Dentist)) OR (oral health professional)) OR (dental hygienist)) OR (dental student)) OR (hygiene student) AND (counseling)) OR (advice)) OR (recommendation)) AND (adults)) OR (adult patients))	436
EBSCO	("HPV vaccination" OR "human papillomavirus vaccine" OR "HPV immunization") AND (dentist OR "oral health professional" OR "dental hygienist" OR "dental student" OR "hygiene student") AND (counseling OR guidance OR advice OR recommendation) AND (adults OR "adult patients")	13
Cochrane Library	HPV:kw AND vaccination:kw AND dentists:kw OR "oral health education":kw AND adults:kw	18
Science Direct	((‘‘Oral health’’ OR ‘‘Dental health’’) AND (‘‘care’’ OR ‘‘prevention’’) AND (‘‘HPV vaccination’’ OR ‘‘Human papillomavirus vaccination’’) AND (‘‘Education’’ OR ‘‘awareness’’ OR Health education)	2330
Wiley	"HPV vaccination OR Human papilomavirus vaccine" anywhere and "Dentists OR oral health professionals" anywhere and "Promotion OR advice OR counseling" anywhere and "Adults OR adult patients" anywhere	4
TRIP database	In the TRIP Database, a structured PICO search was conducted using the terms: (“HPV vaccination” OR “human papillomavirus vaccine” OR “HPV immunization”) AND (dentist OR “oral health professional”) AND (counseling OR guidance OR advice OR recommendation) AND (health education) AND (adults OR “adult patients”)	125
TOTAL		2926

‘‘OR’’ and ‘‘AND’’ are boolean connectors

### Study selection

Study selection was performed in four sequential phases according to PRISMA guidelines:

1. Identification: Database search results collected.
2. Screening: Titles and abstract independently screened by two reviewers (ART and Professor OPL) using the Rayyan platform, excluding studies not meeting eligibility criteria.
3. Eligibility: Full texts of selected studies assessed independently by the same reviewers, with reasons for exclusion documented. Disagreements were resolved by a third reviewer JFB.
4. Included: Studies meeting all criteria were included for qualitative synthesis and meta-analysis.

After selecting and evaluating the 10 included studies, a thematic analysis was performed with the aim of identifying recursive patterns related to the role of the dentist in recommending HPV vaccination. The results and discussion sections of each study were read in full by two reviewers independently. Relevant information was highlighted, coded, and organized into a thematic matrix. The analysis followed an inductive approach, allowing themes to emerge from the study data, without pre-established categories.

Eighteen articles were excluded because they focused primarily on populations under 18 years of age, such as adolescents and children, or on caregivers of these groups. Although caregivers are adults, these studies did not address the adult populations relevant to this review, making them incompatible with its objectives.

Nineteen studies presented a broad focus on vaccination, without specific reference to the HPV vaccine, making it impossible to extract relevant data for this analysis.

Thirteen studies did not specifically focus on the role of OHPs in HPV vaccination, generically addressing other health professionals or contexts unrelated to dental practice.

Eight articles did not include outcomes related to vaccine recommendation, such as intention to recommend, attitudes, or behaviors of OHPs, and were therefore excluded as they did not directly contribute to the objective of this review.

**Fig. 1:**
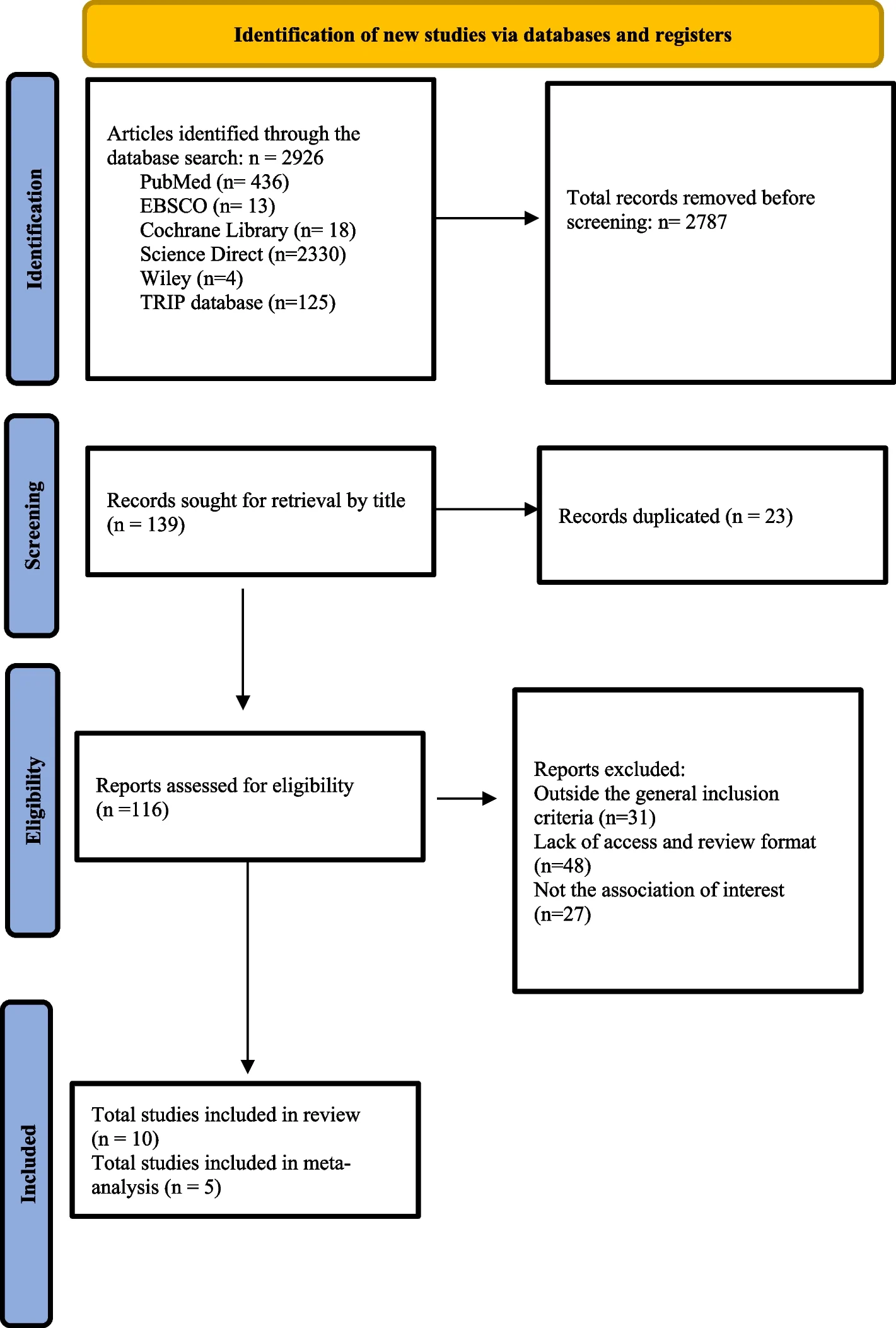
PRISMA (Preferred Reporting Items for Systematic reviews and Meta-Analyses) flow chart of the screening process

A revised PRISMA flowchart (Fig. 1) clearly distinguishes these four phases, specifying 10 studies included in the qualitative synthesis and 5 studies included in the meta-analysis [20].

### Data extraction

Data extraction was conducted independently by two reviewers using a standardized form. Extracted information included: author(s), year of publication, country, study type, participant groups assessed (including ethnicity, age range, and gender), and main outcomes related to HPV vaccination. Additionally, a thematic analysis was conducted on the included studies. Relevant findings concerning the role of OHPs in HPV vaccination counseling were systematically extracted, coded, and categorized to identify consistent patterns, barriers, facilitators, and recommendations.

Relevant findings concerning the role of OHPs in HPV vaccination counseling were systematically extracted, coded, and categorized, distinguishing between observational studies [21–25] and those evaluating interventions [26–30].

### Risk of bias in individual studies

The methodological quality of included studies was assessed using the JBI Critical Appraisal Checklist for Analytical Cross-Sectional Studies [31, 32]. Each article was rated as ‘‘yes’’, ‘‘no’’ and ‘‘uncertain’’ for each criterion. Results are presented in Table 2 (detailed checklist scores) and Table 3 (a visual traffic light plot) to enhance clarity and reader interpretation. Both tables represent the same evaluations using this single tool.

**Table 2:** Critical assessment of methodological quality – JBI critical appraisal checklist for analytical cross-sectional studies

**Question/article**	Kline et al., [27] 2018	Shukla et al., [30] 2018	Pampena et al., [28] 2019	Patel et al., [25] 2019	Salous et al., [29] 2020	Guadiana et al., [26] 2021	Askelson et al., [24] 2023	Merrell et al., [23] 2023	Naavaal et al., [22] 2023	Steinbaum et al., [21] 2023
1. Were the criteria for inclusion in the sample clearly defined?	Yes	Yes	Yes	Yes	Yes	Yes	Yes	Yes	Yes	Yes
2. Were the study subjects and environment described in detail?	Yes	Yes	Yes	Yes	Yes	Yes	Yes	Uncertain	Uncertain	Uncertain
3. Was the exposure measured validly and reliably?	Yes	Yes	Yes	Uncertain	Yes	Yes	Yes	Yes	Yes	Yes
4. Were objective and standardized criteria used to measure the condition?	No	Yes	Yes	Yes	No	Yes	No	No	No	Yes
5. Were confounding factors identified?	Uncertain	No	No	Yes	No	Yes	Yes	Uncertain	No	No
6. Have strategies been stated to deal with confounding factors?	No	No	No	Uncertain	No	No	No	No	No	No
7. Were the results measured in a valid and reliable way?	Yes	Yes	Yes	Yes	Yes	Yes	Yes	Yes	Yes	Yes
8. Was appropriate statistical analysis used?	Yes	Yes	Yes	Yes	Yes	Yes	Yes	Yes	Yes	Yes
Percentage	62.50%	75.00%	75.00%	75.00%	62.50%	87.50%	75.00%	50.00%	50.00%	62.50%
Risk	Moderate	Low	Low	Low	Moderate	Low	Low	Moderate	Moderate	Moderate

Values expressed as percentage (%)

**Table 3:** Traffic light plot illustrating the risk of bias assessment using the National Institutes of Health (NIH) quality assessment tool

Article	Q1	Q2	Q3	Q4	Q5	Q6	Q7	Q8	Overall
Kline et al., [27] 2018									5
Shukla et al., [30] 2018									6
Pampena et al., [28] 2019									6
Patel et al., [25] 2019									6
Salous et al., [29] 2020									5
Guadiana et al., [26] 2021									7
Askelson et al., [24] 2023									6
Merrell et al., [23] 2023									4
Naavaal et al., [22] 2023									4
Steinbaum et al., [21] 2023									5

The colors green, yellow, and red represent low, unclear, and high risk of bias respectively in each domain/question of the included studies

Q1: Were the criteria for inclusion in the sample clearly defined?

Q2: Were the study subjects and environment described in detail?

Q3: Was the exposure measured validly and reliably?

Q4: Were objective and standardized criteria used to measure the condition?

Q5: Were confounding factors identified?

Q6: Have strategies been stated to deal with confounding factors?

Q7: Were the results measured in a valid and reliable way?

Q8: Was appropriate statistical analysis used?

### Data analysis

Data analysis was performed using RStudio (version 4.2.2). A random-effects model was applied to calculate the pooled proportion and 95% confidence interval (CI) for all analyzed outcomes. The meta package in R was used to apply the metaprop function.

Heterogeneity among included studies was assessed using the I^2^ statistic, τ^2^, and Cochran’s Q test. The I^2^ statistic quantifies the percentage of total variation between studies due to heterogeneity rather than chance. Values of I^2^ below 40% were considered low, 40–75% moderate, and above 75% substantial heterogeneity. These thresholds guided interpretation and model selection but were considered in the context of clinical and methodological diversity across studies.

In our analysis, the pooled estimates showed considerable heterogeneity, with I^2^ values ranging from 0 to 97%. For example, in the analysis of barriers to HPV vaccination, subgroups such as “Lack of knowledge” (I^2^ = 97%, *p* < 0.001), “Privacy concerns” (I^2^ = 95%, *p* < 0.001), and “Not comfortable” (I^2^ = 96%, *p* < 0.001) showed particularly high heterogeneity. Conversely, the subgroup “Religious reasons” showed no observed heterogeneity (I^2^ = 0%, *p* = 0.82). Overall heterogeneity across studies was also high (I^2^ = 98%, *p* < 0.001). Similar variability was observed in the knowledge and attitudes section, with I^2^ values ranging from 61 to 95%.

These findings justified the use of a random-effects model with a random-intercept logistic regression approach and maximum-likelihood estimation for τ^2^, which appropriately accounts for between-study variability. Despite heterogeneity, meta-analytic pooling of proportions has been widely used in public health research to summarize patterns across diverse observational studies. In this review, the meta-analysis is intended to provide a pooled estimate of general trends rather than a precise effect size. Consequently, the narrative synthesis is employed as the primary tool for interpretation, ensuring that the nuances of different study contexts and qualitative patterns are fully captured alongside the statistical estimates.

### Assessment of publication bias

Potential publication bias was assessed through visual inspection of a funnel plot generated from the proportion estimates and standard errors of the studies included in the meta-analysis. Egger’s linear regression test for funnel plot asymmetry was also performed to evaluate the presence of small-study effects.

Given the variation in study outcomes and participant perspectives, the funnel plot should be interpreted with caution, as outcome heterogeneity may confound the visual symmetry typically used to assess publication bias. Specifically, when statistical heterogeneity is high, the included studies likely represent distinct populations or methodological frameworks rather than a single ‘‘true’’ effect. In such instances, an asymmetrical distribution in the funnel plot may arise from these inherent clinical and methodological differences between studies, rather than indicating the presence of publication bias alone.

## Results

**Fig. 2:**
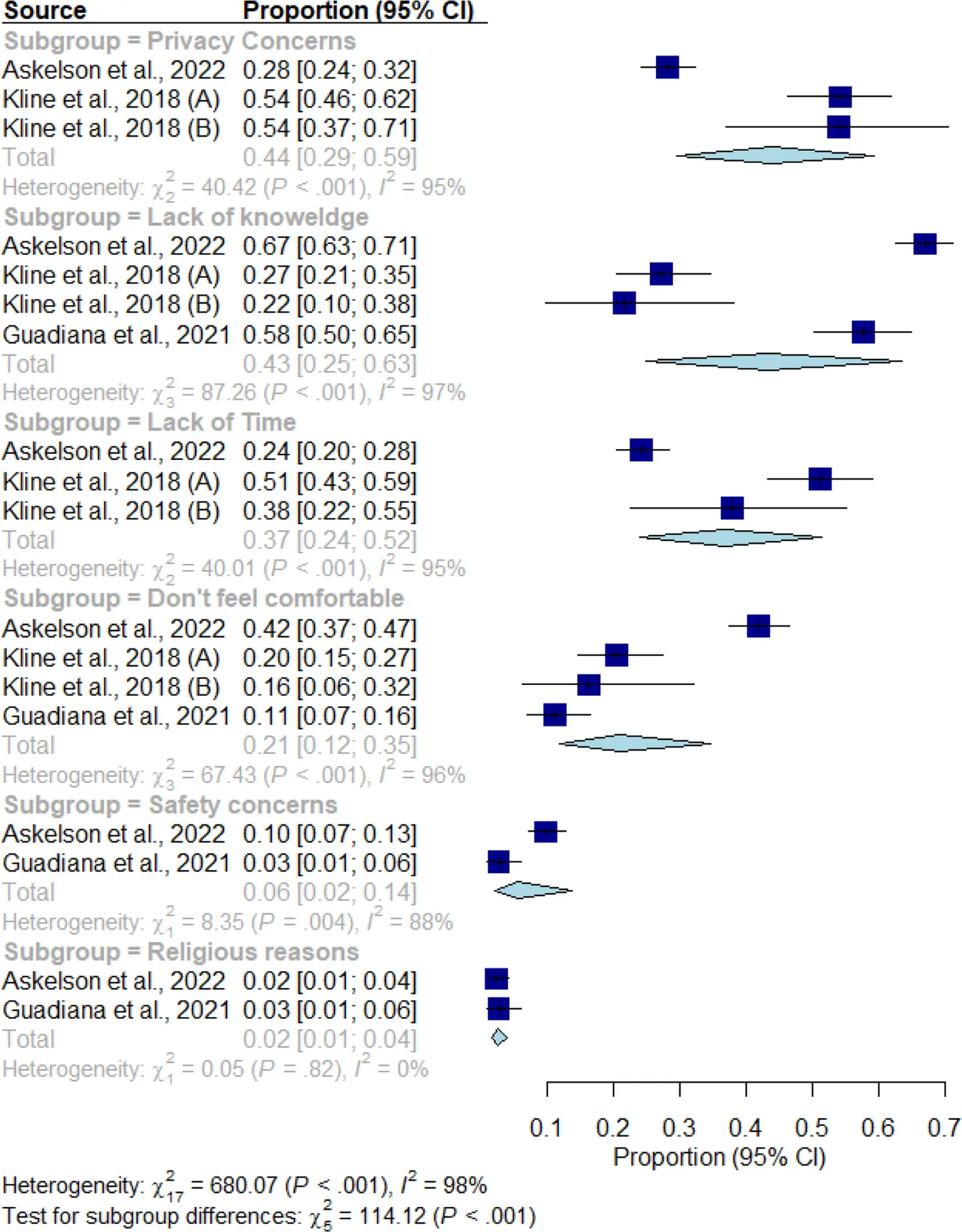
Prevalence of barriers among dental professionals. Barriers include lack of privacy (44%), limited knowledge (43%), time constraints (38%), and discomfort discussing HPV (11%), with corresponding 95% confidence intervals

**Fig. 3:**
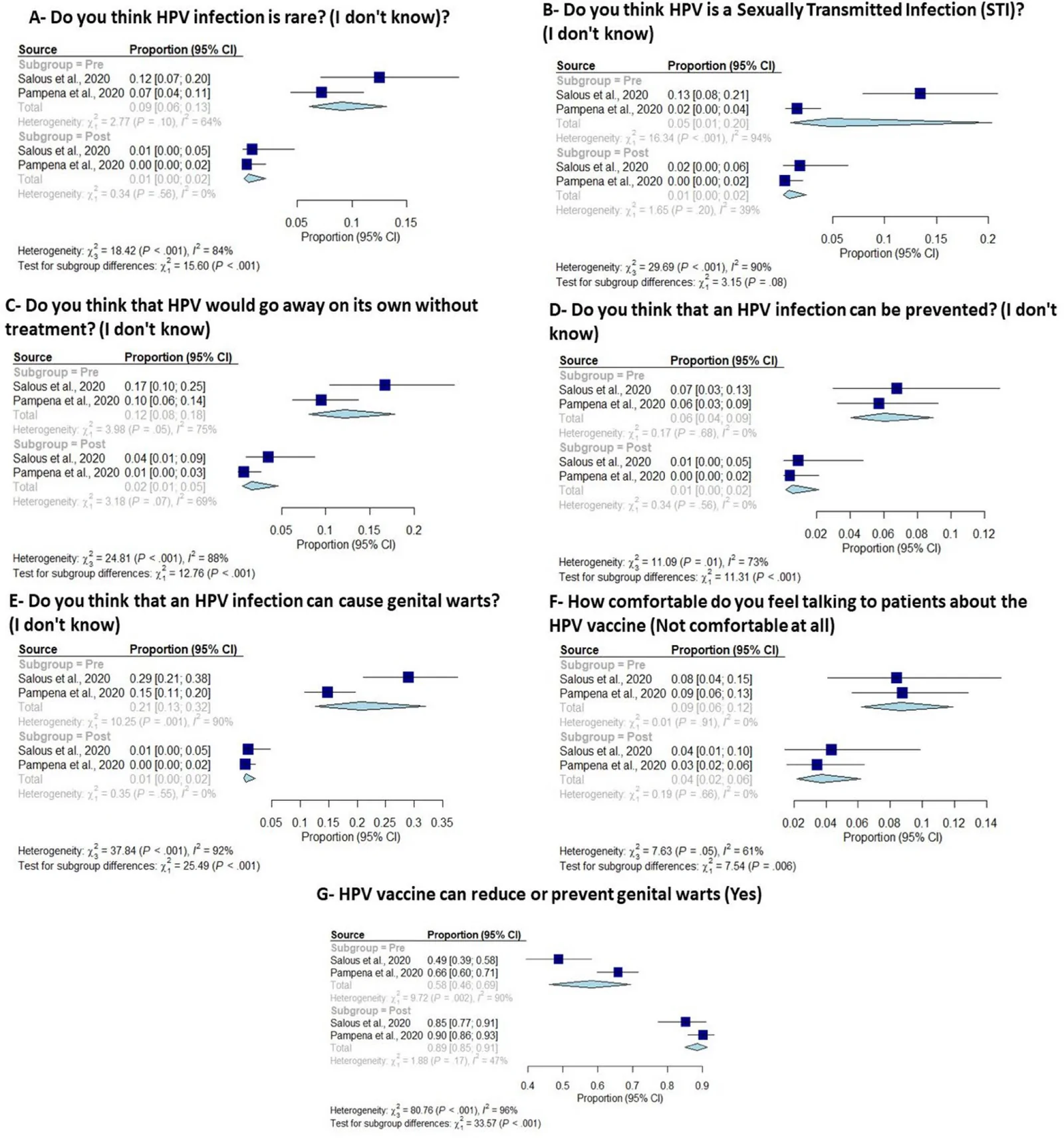
The change in knowledge among dental professionals pre and post-questionnaires. The dark blue squares represent the prevalence in each study while the light blue diamond represents the pooled prevalence

Searches of databases and other sources identified 2926 records. After removing 2787 irrelevant articles, 139 articles remained for further review. Twenty-three duplicates were removed, leaving 116 records for eligibility assessment. Of these, 31 were excluded for not meeting the general inclusion criteria, 48 due to lack of access or review format, and 27 not addressing the association of interest. A total of ten studies were included in the review (Fig. 1), of which five were part of the meta-analysis (Figs. 2 and 3).

All included studies were conducted in the United States, covering diverse geographic regions such as New England, Arizona, Michigan, Iowa and Pennsylvania, as well as specific populations including American Indian and Alaska Native communities.

Most studies recruited OHPs, including dentists, hygienists, and dental students. Only three studies included other groups: adults aged ≥ 18 years from the general population or university students enrolled in health-related courses [21–23]. In these cases, inclusion served to assess the adult public’s knowledge, attitudes, and willingness to receive HPV vaccination recommendations from OHPs, as well as the future intentions of students who may later enter clinical practice. Specifically, Steinbaum et al. [21] examined adult patient’s attitudes toward HPV vaccination and their perceptions of the dentist’s role in vaccine advocacy. Naavaal et al. [22] assessed knowledge and perceptions about HPV and preventive counseling among adults aged ≥ 18 years. Merrell et al. [23] focused on the comfort levels of university students enrolled in a health-related course future OHPs when discussing HPV vaccination. In these three studies, a purely observational cross-sectional study was conducted, without any educational intervention or training component.

Participants’ ages ranged from 18 to 75 years, though most fell between 30 and 60 years. Studies involving dental students typically included younger participants (18–19 years) [23, 26]. Across studies, the majority of participants were female. Notably, all dental hygienists in the included samples were women [24, 27]. This reflects current workforce demographics and may be relevant given that women are more likely to have been vaccinated and to possess higher awareness of HPV-related risks, partly due to historical public health campaigns focused on cervical cancer prevention.

Ethnicity data, when reported, indicated that most participants were Caucasian, followed by African American, Asian and Hispanic individuals. Some studies targeted specific populations, such as Alaska Natives and American Indians [28, 29]. For example, Salous et al. [29] examined OHPs from Native American and Alaska Native communities, assessing their knowledge and comfort levels in discussing HPV vaccination, while Pampena et al. [28] evaluated the impact of educational interventions in these populations. These studies provide valuable insights into cultural and regional differences in vaccination-related counseling.

Across the ten studies, several key themes emerged regarding the role of OHPs in HPV vaccination counseling. Awareness of the link between HPV, oral health, and oropharyngeal cancer risk was limited among both patients and professionals. In North America, only 32.8% of adults considered the HPV vaccine ‘‘very important’’ for disease prevention a proportion higher among those aware of its link to oral health. In Steinbaum et al. [21], 93% of respondents reported being informed about COVID-19 vaccination, compared to 50% for HPV vaccination. Just half of participants knew that HPV vaccination could prevent certain head and neck cancers. Comfort in receiving vaccine recommendations from OHPs varied by ethnicity, with Caucasian individuals feeling more comfortable accepting clinical vaccination recommendation from OHPs compared to African American individuals (Table 4).

**Table 4:** Summary of study characteristics, participant demographics, and key findings on the role of oral health professionals in HPV vaccination

Author, year	Type of study	Country	Groups	Ethnicity	Age	Gender	Scope of practice	Private vs public practice	Main results
Kline et al., [27] 2018	Mixed-method with focus groups and surveys	USA	• Dentists in focus groups *n* = 33 • Hygienists in focus groups *n *= 48 • Dentists in surveys *n* = 37 • Hygienists in surveys *n* = 166	The majority of participants were of Caucasians ethnicity followed by African American and Asian	•Dentists in focus groups average age 46.9 years •Average age of research dentists 47.8 years •Dental hygienists in focus groups average age 45.7 years •Dental hygienists surveyed average age 40.4 years	More than half of the participants in the study are female, with (99%) of the hygienists and (54%) of the dentists	Dentists in focus groups 19.2 •Hygienists in focus groups 20.7 •Dentists in surveys 19.8 •Hygienists in surveys 15.1	Most participants worked in the private sector	OHPs identified a need for more education and awareness to facilitate HPV-related discussions. Dental hygienists focused on prevention, while dentists emphasized secondary prevention. Main barriers included lack of privacy (54%) and fear of offending patients (51%). Participants requested passive educational tools like pamphlets and videos
Shukla et al., [30] 2018	Pre-post study educational intervention	USA, New England	•Dentists (44.2%) •Oral hygienists (42.3%)	•Caucasian *n* = 43 (53.9%) •Asian *n* = 13 (14.6%) •African-American *n* = 13 (14.6%) •Hispanic *n* = 11 (12.4%)	Between 55–75 years old *n* = 26/49 (53.1%)	♀ = 36 (73.5%) ♂ = 13 (26.5%)	Did not inform the participant’s years of practice	• 59.2% private practice • 31.6% public practice • 9.2% research	An educational intervention significantly improved professional’s comfort (from 26.2% to 67.5%) and perceived readiness to discuss HPV. After the intervention, 91.6% reported increased HPV knowledge and 37.5% reported more patient discussions. 82.6% acknowledged their role in patient education
Pampena et al., [28] 2019	Pre-post study educational intervention	USA, New England	• Dentists *n* = 69 (26.6%) •Hygienists *n* = 131 (50.6%)	•Caucasian *n* = 213 (84.9%) •Asian *n* = 21 (8.4%) •African-American *n* = 9 (3.6%) • Alaska Native *n* = 5 (2%)	•20–30 years (16.1%) •31–40 years (25.7%) •41–50 years (23.4%) •51–60 years (21.1%) • > 60 years (13.8%)	♀ = 156 (82.5%) ♂ = 33 (17.5%)	Did not inform the participant’s years of practice	Most participants worked in the private sector	Post-intervention surveys showed an increase in knowledge about HPV, including its relationship to genital herpes and oropharyngeal cancer. Belief in vaccine efficacy increased from 66.7% to 88.3%. No significant differences in knowledge were found between dentists and hygienists
Patel et al., [25] 2019	Cross-sectional study	USA, Arizona	• Dentists *n* = 257 (36.15%) •Hygienists *n* = 454 (63.85%)	• Caucasian (78.5%) • Hispanic (10.6%) • Asian (4.9%) • African American (1.7%)	*n* = 307 (43.6%) of the participants were between 35–54 years old	♀ = (73.1%)	• Dentists: 21 years of practice • Hygienists: 14 years of practice	Did not inform whether the participants worked in the public or private sector	Both dentists and hygienists expressed willingness to educate patients. 49% of dentists and 42.4% of hygienists spent 5–9 min per patient on education. Dentists were more likely to refer patients for vaccination and less concerned about vaccine safety. Both groups supported training programs
Salous et al., [29] 2020	Pre-post study educational intervention	USA, American Indian and Alaska Native Population	•Dentist *n* = 54 (45%) • Hygienist *n* = 21 (17.5%) •Dental assistant *n* = 36 (30%)	• Caucasian *n* = 49 (41.2%) • Alaska Native *n* = 38 (31.9%) •African- American *n* = 3 (2.5%) • Asian *n* = 6 (5%)	Participants between 31–60 years of age in (72%) of cases	♀ = (64%) ♂ = (36%)	Did not inform the participant’s years of practice	Did not inform whether the participants worked in the public or private sector	Knowledge about HPV and its sexual transmission improved after educational efforts. Only 16.2% had prior training, and 70.1% cited lack of knowledge as a barrier to patient discussions. Preferred learning methods included in-person (64.1%) and webinars (61.5%)
Guadiana et al,. [26] 2021	Cross-sectional study	USA, Michigan	• Dentists *n* = 140 (22%) •Hygienists *n* = 91 (91%) •Dental medicine students *n* = 242 (24%) •Hygiene students *n* = 150 (23%)	No information in the article	Participants between 19–72 years of age, with an average of 32.6 and median of 26	♀ = 407 participants (65%) ♂ = 216 participants (35%)	• 40% of dentists had more than 25 years of practice • 43% of hygienists had more than 25 years of practice	Did not inform whether the participants worked in the public or private sector	Willingness to administer the HPV vaccine was highest among students. Confidence discussing HPV was generally low but improved with prior training. The need for additional training (58%) and liability concerns (30%) were main barriers to administering the vaccine
Askelson et al., [24] 2023	Mixed-methods study with a cross-sectional survey and interviews	USA, Iowa	•Approximately 597 hygienists responded to the survey, eliminating those who did not work in private practice. Thus, 417 hygienists responded to the survey, with another 19 who were interviewed • 20 dentists were interviewed	No information in the article	In surveys of hygienists, approximately (48.5%) are between 40–59 years old and (37.4%) are between 27–39 years old In the interviews, (63.2%) of hygienists were between 40 and 59 years old. However, (40%) of dentists were between 27 and 39 years old and over 60 years old	In the interviews, males scored 60% in the area of ​​dentistry While the female sex has a prevalence of 100% in the area of ​​oral hygienist	Did not inform whether the participants worked in the public or private sector	The focus was on OHPs working in the private sector, with 127 participants working in public practices being eliminated	Barriers to HPV vaccine promotion included lack of knowledge (67%), discomfort (41.9%), and perceived scope limitations (31.5%). Only 25.6% received HPV-specific training, yet 87.2% were willing to learn more. Some hygienists questioned vaccine safety (9.8%)
Merrell et al., [23] 2023	Mixed-methods study using an online survey with qualitative analysis of open-ended responses	USA	• College students enrolled in an introductory health course at a large public university in the Mid-Atlantic region of the United States	• Caucasian (73.1%) • African American (6%) • Hispanics (2.1%)	Approximately (76.4%) of participants were aged between 18–19 years	♀ = (71%)	Not applicable	Not applicable	Participants were more comfortable discussing general prevention than HPV. Only 6.3% wanted dentists to talk about HPV. Reluctance stemmed from lack of awareness (74.1%) and perception of the topic as personal or taboo
Naavaal et al., [22] 2023	Cross-sectional study	USA	•Target population, individuals ≥ 18 years old	•Caucasian (62.8%) •Hispanic (16.7%) •African American (12%) • Asian (4.8%)	• 18–29 years (20.7%) • 30–44 years (25%) • 45–59 years (24.3%) • ≥ 60 years (30%)	♀ = (51.5%) ♂ = (48.5%)	Not applicable	Not applicable	32.8% viewed the HPV vaccine as very important, and 56.7% believed OHPs were qualified to educate patients. Knowledge was linked to higher vaccine acceptance and trust in OHPs. Women and minorities were less likely to perceive dentists as HPV educators
Steinbaum et al., [21] 2023	Cross-sectional study	USA, Pennsylvania	•Target population, individuals ≥ 18 years old	•Caucasian (32%) •African-American (44%) •Hispanic (7%)	18–45 years old, with an average of (31.61%)	♀ = 109/158 (67%)	Not applicable	Not applicable	While 61% of participants saw dentists as qualified to administer COVID-19 vaccines, only 52% felt the same about HPV. Awareness of HPV was lower than for COVID-19. Caucasians were more likely to trust oral health professional’s vaccine recommendations than African Americans

(%) Values ​​expressed as percentage

♀ feminine ♂ masculineHPV: Human papillomavirusCOVID-19: coronavirus disease 2019

### Barriers to counseling

Data on barriers to discussing HPV vaccination were extracted from all studies reporting them, then grouped by conceptual similarity. Pooled meta-analytic estimates were calculated when outcome definitions and response formats were comparable. The most common barrier was lack of privacy in the clinical setting (44%, 95% CI [37%—71%]), followed closely by limited HPV-related knowledge (43%, 95% CI [25%—63%]). Other barriers included time constraints (38%, 95% CI [22%—55%]), discomfort discussing the topic (11%, 95% CI [7%—16%]), safety concerns (3%, 95% CI [1%—6%]), and religious objections (2%, 95% CI [1%—4%]) (Fig. 2).

### Educational interventions

Of the ten studies included in this review, only three specifically evaluated educational interventions targeting OHPs [28–30]. These interventions, delivered through lectures, workshops or digital modules, aimed to improve knowledge about HPV, its transmission, and the role of vaccination in cancer prevention. Pre and post-intervention assessments consistently demonstrated improvements in participants’ knowledge, comfort, and willingness to discuss HPV vaccination with patients.

In these studies, OHPs answered a questionnaire before the intervention and a post-lecture questionnaire to assess whether the training improved their knowledge. For example, regarding the first question, “*Do you think HPV infection is rare?*”, the percentage of those who answered ‘‘I don’t know’’ decreased from 9% prior to the intervention to 1% after the intervention. For the question “*Do you think HPV is a sexually transmitted infection?*”, those who answered ‘‘I don’t know’’ decreased from 5 to 1%. Similarly, for the question “*Do you think that HPV would go away on its own without treatment?*”, the percentage of those who answered ‘‘I don’t know’’ decreased from 12 to 2%.

For the question “*Do you think that an HPV infection can be prevented?*”, the percentage of those who answered "I don’t know" decreased from 6 to 1%. Additionally, for the question “*Do you think that an HPV infection can cause genital warts?*”, the percentage of those who answered "I don’t know" decreased significantly from 21 to 1%.

Moreover, for the question “*How comfortable do you feel talking to patients about the HPV vaccine?*”, the percentage of those who answered ‘‘not comfortable at all’’ decreased from 9 to 4%.

Finally, for the question “*HPV vaccine can reduce or prevent genital warts?*”, the percentage of those who answered ‘‘Yes’’ increased from 58 to 85%.

The meta-analysis of the pre- and post-intervention data regarding professional knowledge is summarized in Fig. 3, illustrating the shift in the prevalence of correct responses across the evaluated studies (Fig. 3).

**Fig. 4:**
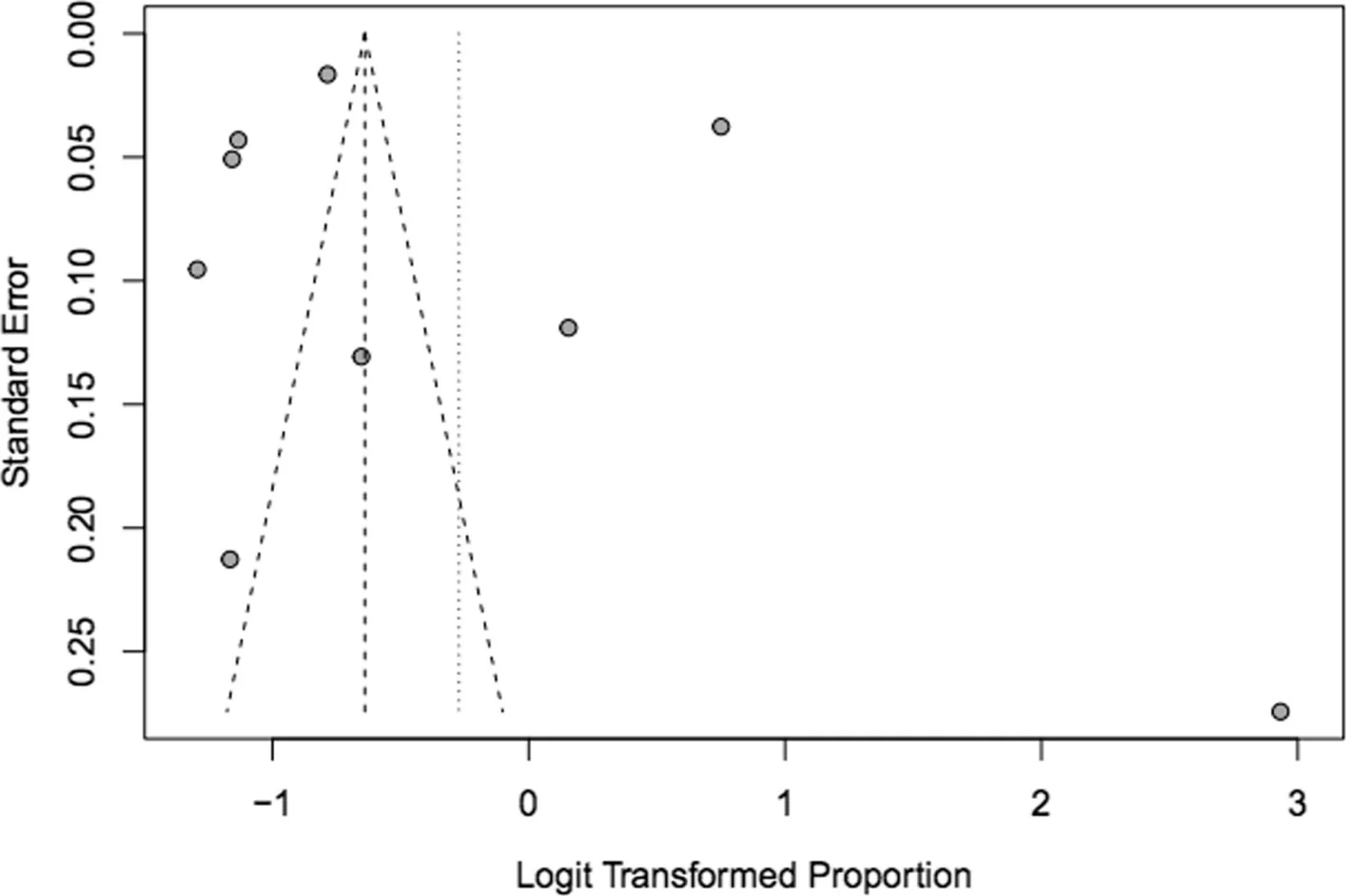
Funnel plot, with no evidence of publication bias (*p* = 0.6299), with residual heterogeneity estimated at tau^2^ = 267.3897. Inverse variance weighted studies

Potential publication bias was assessed through visual inspection of a funnel plot generated from the proportion estimates and standard errors of the nine studies included in the final analysis (Fig. 4). One study (Steinbaum et al. [21]) was excluded from the funnel plot due to insufficient data to calculate a valid event proportion. Egger’s linear regression test for funnel plot asymmetry produced a t-statistic of 0.50 with 7 degrees of freedom and a p-value of 0.6299. The estimated bias was 4.0606 (SE = 8.0604) and the multiplicative residual heterogeneity variance (τ^2^) was 267.3897.

Figure 4 presents the funnel plot with these parameters.

## Discussion

### Risk of bias evaluation

The methodological quality of the included studies varied, with five out of ten articles assessed as having a moderate risk of bias, mainly due to the absence of standardized measurement criteria, insufficient description of participants and settings, and lack of control for confounding factors [21–23, 27, 29]. For example, Kline et al. [27] did not use objective criteria to measure outcomes or address confounders clearly. In contrast, the other five studies showed low risk of bias, indicating higher reliability and relevance [24–26, 28, 30].

All studies were cross-sectional, which limits causal inference. The absence of control groups and inconsistent management of confounding variables further restricts internal validity. Additionally, the predominance of studies conducted in the United States limits the generalizability of findings to other geographic or cultural contexts.

Regarding publication bias, the visual inspection of the funnel plot and Egger’s test (*p* = 0.6299) indicated no significant evidence of publication bias, suggesting that the results are unlikely to be influenced by small-study effects. However, these findings should be interpreted with caution. The substantial heterogeneity observed across study outcomes and participant perspectives may confound the visual symmetry typically used to assess publication bias.

Furthermore, the high statistical heterogeneity (I^2^ = 98%) represents an important limitation. Methodological variations – such as differences in study design, target populations, and data collection tools – limit the generalizability of the findings. Consequently, the meta-analytic estimates should be regarded as indicative of overall trends rather than precise effect sizes, reinforcing the importance of the narrative synthesis for a comprehensive interpretation of the role of OHP in HPV vaccination.

### Barriers to HPV vaccination counseling among oral health professionals

This review reveals several significant barriers faced by OHPs when counseling adults on HPV vaccination. The most frequently cited obstacles include lack of privacy in dental offices (reported by 44% of participants) and limited knowledge regarding HPV and its vaccination (67%). These findings underscore the need for multifaceted interventions addressing both the physical environment and the educational preparation of OHPs.

Educational interventions have demonstrated positive effects, with training programs increasing dentist’s confidence to discuss HPV vaccination from 26.2% feeling prepared before intervention to 67.5% after [30]. This suggests that targeted training can effectively reduce knowledge gaps and discomfort when approaching this sensitive topic. Together, these findings indicate significant knowledge deficits among OHPs regarding HPV prior to training. However, the results also demonstrate the effectiveness of targeted educational interventions in addressing these deficits, as evidenced by the consistent improvement in knowledge scores and counseling confidence.

Limited knowledge and communication difficulties likely stem from insufficient coverage of HPV-related content during dental education and continuing professional development. Integration of digital tools such as interactive modules, explanatory videos, and simulated counseling scenarios has shown promise to enhance both knowledge and communication skills in an accessible, scalable manner [27, 28, 30]. The success of similar digital training in other health fields (e.g., cardiovascular emergency training for nurses) supports the potential of this approach [33–35].

### Influence of practice setting and sociocultural factors

The type of dental practice influences counseling practices. Dentists working in private settings report greater discomfort discussing HPV vaccination compared to those in public clinics, where institutional support and public health campaigns facilitate communication [24, 27, 30, 36]. In countries like Portugal, where HPV vaccination for adults is not subsidized, dentist’s advisory role becomes even more crucial, given their frequent contact with adult patients.

Cutural and regional differences also affect the implementation and effectiveness of vaccination counseling. Most studies originate from the USA, limiting applicability to other contexts with different sociocultural dynamics and health systems. Future research should explore these factors to tailor interventions accordingly.

### Translating findings into practical strategies

Addressing the identified barriers requires comprehensive, multifaceted strategies. Creating private spaces within dental offices could enhance patient comfort and confidentiality, fostering more open discussions about HPV vaccination [37]. Integrating dentists into national vaccination campaigns would provide institutional backing and increase public awareness, legitimizing their role as vaccination advocates [38]. Expanding continuing education through accessible digital platforms would allow OHPs to acquire up-to-date knowledge and develop effective communication skills flexibly and efficiently [38].

International examples illustrate the benefits of such approaches. Countries like Australia and the United Kingdom have successfully involved dentists in vaccination policies and campaigns, leading to improved vaccine uptake and increased public awareness [37]. In these countries, dentists are encouraged to participate in HPV vaccination awareness campaigns. While vaccine administration by dentists is not yet widespread, they play a critical role in patient education and vaccination recommendation [39]. These models demonstrate the potential gains from policy support, educational investment, and system-level integration of OHPs into broader public health initiatives.

### Expanding the role of dental professionals in HPV vaccination promotion

The role of OHP, particularly dentists, in promoting HPV vaccination represents an important frontier in integrating dentistry with broader public health strategies. Traditionally, dentists have focused on clinical and curative aspects of oral health, but the increasing recognition of the link between HPV and oropharyngeal cancers is expanding their scope to include primary prevention, especially through vaccination counseling. This expanded role requires not only changes in initial academic training but also robust investment in continuing education to ensure professionals are equipped with up-to-date knowledge and confidence. While approximately 28.2 million Americans visit a dental setting without a concurrent medical visit – creating a ‘‘unique opportunity’’ for public health outreach – current practice patterns reveal significant gaps. Evidence suggests that OHPs do not always provide recommendations based on risk profiles; for instance, adults without traditional oral cancer risk factors are sometimes more likely to receive counseling or screenings than high-risk individuals [11]. This inconsistency highlights that merely having access to patients is insufficient. OHPs require systematic training and institutional support to transition from curative care to proactive HPV vaccination advocacy.

Continuing education is key to empowering dentists to take on additional responsibilities in HPV vaccination counseling and promotion. Multiple studies highlight significant knowledge gaps regarding HPV and vaccination among OHPs, which directly affect their confidence and willingness to discuss the topic with patients [27, 30, 35].

From an ethical perspective, expanding the dentist’s role in HPV vaccination promotion aligns with fundamental bioethical principles, particularly beneficence and justice. By providing accurate information and appropriate recommendations, dentists contribute to protecting individual and public health by preventing serious diseases such as oropharyngeal cancer, which significantly impact morbidity and mortality [27, 33]. Additionally, dentists’ involvement may help reduce health disparities, especially in vulnerable populations with limited access to immunization information and services [30, 35]. As active public health agents, OHPs can identify at-risk patients, offer personalized counseling, and refer them for vaccination, thereby increasing vaccine coverage.

However, practical implementation faces challenges, including structural limitations such as lack of adequate private space for confidential vaccination discussions, as well as cultural barriers and varying perceptions among patients and professionals [39]. These issues highlight the need for integrated strategies involving professionals, institutions, public policies, and health campaigns to create an environment conducive to vaccination promotion within dentistry [30, 38]. Policies encouraging and regulating dentist’s roles in vaccination campaigns, including potential vaccine administration in dental offices, could strengthen this practice and enhance its impact [37].

It is also important to consider regional and sociocultural differences in HPV vaccination approaches. Most studies in this review originate from the United States and reflect specific contexts that may not be directly transferable to other settings, such as Portugal and European countries. Interventions should be adapted to reflect local cultural, economic, and healthcare system particularities to ensure effectiveness and acceptance [40, 41]. This adaptation should also consider patient age, socioeconomic status, gender, and personal beliefs, which significantly influence vaccine acceptance.

The dentist’s role in HPV promotion also aligns with a growing trend toward integrated, multidisciplinary healthcare that values collaboration among different professionals to improve health outcomes. Dentists can partner with physicians, nurses, pharmacists, and others to form support networks facilitating patient access to information, vaccination, and follow-up care. This collaborative approach is especially relevant for diseases like oropharyngeal cancer, which require multifaceted preventive strategies.

### Strengths, limitations and future research directions

A key strength of this review is the implementation of a meta-analysis as an exploratory synthesis tool. Rather than seeking a single, definitive universal effect size, the meta-analysis provides a quantitative map of the existing evidence and illustrates the extent of its variability within the dental setting ecosystem. The use of forest plots serves as a powerful visual aid to highlight the divergence in outcomes among different populations, which is, in itself, a significant finding of this research.

This review is subject to several limitations. The predominance of cross-sectional studies limits the ability to draw causal conclusions and hinders to assessment of long-term intervention effects. The heterogeneity of study populations including practicing dentists, other OHPs, and students as well as the variety of intervention types (face-to-face, digital, or blended) complicate result synthesis and identification of best practices.

These limitations, including the heterogeneity of study populations and interventions, the predominance of cross-sectional designs, and methodological variability, were explicitly acknowledged and carefully considered in our interpretation of results. Consequently, our conclusions should be regarded as indicative of overall trends and gaps in the evidence rather than as precise effect estimates.

The high heterogeneity observed in the analyses, particularly in the pooled data on barriers faced by OHPs, represents an important limitation of this review. Methodological variations among the included studies – such as differences in study design, target populations, data collection tools, and how barriers were categorized—may have contributed to this heterogeneity. For example, although lack of privacy was identified as the most common barrier (44%, 95% CI [37% – 71%]), the wide confidence interval indicates substantial variability across studies. This heterogeneity limits the generalizability of the findings and underscores the need for standardization in future studies evaluating the role of OHPs recommending HPV vaccination. Therefore, results should be interpreted with caution, taking these contextual and methodological differences into account.

Future research should prioritize longitudinal designs to evaluate lasting impacts, focus on clinical realities with attention to differences between public and private settings, investigate policy measures to more actively involve dentists in vaccination efforts, and assess the feasibility and impact of vaccine administration within dental offices. It is also important to evaluate both digital and in-person training interventions using standardized and validated outcome measures [35]. Such research will strengthen the evidence supporting the role of dentists as public health agents in promoting HPV vaccination.

The high statistical heterogeneity observed in our analyses (with I^2^ values reaching 98%) underscores the diverse nature of the evidence currently available on this topic. As a result, the findings from our meta-analysis should be interpreted as indicative trends within the dental setting, rather than absolute values. To address this, we have reinforced the narrative synthesis as our primary interpretative framework, allowing for a more comprehensive understanding of how barriers and educational interventions vary across different populations and geographic regions, which a single pooled estimate might otherwise obscure.

Moreover, systematic evaluation of both digital and traditional educational interventions using standardized and validated outcome measures is essential. Such research will generate robust evidence to support the integration of dentists as active agents in public health efforts to increase HPV vaccination coverage.

## Conclusion

The results of this study highlight the strategic role of dentists in promoting HPV vaccination, especially in a context of increasing rates of oropharyngeal cancer associated with the virus. Despite the great potential of these professionals in raising patient awareness, barriers such as lack of privacy, specific training and discomfort in approaching sensitive topics make effective counseling difficult. To overcome these challenges, it is crucial to implement targeted educational interventions and promote environments that encourage open discussions about HPV. By addressing these barriers, dentists can play a key role in promoting vaccination and ultimately reducing the burden of HPV-related cancers. As previously noted, the predominance of studies from the United States and the heterogeneity of the analyzed populations may limit the generalizability and certainty of some findings. Future research should prioritize context-specific interventions and explore the direct integration of OHPs into vaccination strategies, particularly across diverse public and private healthcare settings.

## Supplementary Information

Supplementary Material 1.

## Data Availability

No datasets were generated or analysed during the current study.

## Abbreviations

Covid-19: Coronavirus disease 2019
HPV: Human papillomavirus
OHPs: Oral Health Professionals
PICO: Population, Intervention, Comparison and Outcome
PRISMA: Preferred Reporting Items for Systematic Review and Meta-Analysis
WHO: World Health Organization
